# A Cross-Sectional Study Assessing the Clinical Traits of Patients With Dengue

**DOI:** 10.7759/cureus.79182

**Published:** 2025-02-17

**Authors:** Shubhransu Patro, Arushi Choudhary, Soumayan Mondal, Vibha Sharma, Chikkam Sandeep, Sailendra Nayak, Jyoti Prakash Sahoo

**Affiliations:** 1 General Medicine, Kalinga Institute of Medical Sciences, Bhubaneswar, IND; 2 Pharmacology, Kalinga Institute of Medical Sciences, Bhubaneswar, IND

**Keywords:** c-reactive protein level, dengue with warning signs, gallbladder wall thickening, hepatosplenomegaly, immature platelet fraction, ns1 antigen, pleural effusion, serum lactate dehydrogenase, severe dengue fever, total platelet count

## Abstract

Background and objectives: Dengue fever has been prevalent in tropical and subtropical countries since the beginning of this century. Though its clinical features and impacts on various organs have been explored thoroughly, we still lack a concrete correlation among those traits. Hence, we carried out this study to evaluate various clinical parameters of patients with dengue infection. Moreover, we planned to determine the co-occurrence of the symptoms and correlate all clinical parameters.

Methods: This cross-sectional study ran from January to December 2024 at the Kalinga Institute of Medical Sciences (KIMS), Bhubaneswar, India. We enrolled adult dengue patients admitted to the KIMS medicine ward last year with a positive non-structural 1 protein (NS1) report. We slotted the participants into the following groups: severe dengue and dengue with and without warning signs. The warning signs are continuous vomiting, rapid decline in the platelet count, hepatomegaly > 2 cm, mucosal bleeding, and fluid accumulation. Severe dengue infection is often presented with either of the following symptoms: respiratory distress, impaired consciousness, shock, elevated liver enzymes, and severe bleeding. From their case sheets, we noted their clinical (e.g., hematological, hepatic, and renal) parameters during admission. In the study population, we evaluated the incidences of pleural effusion, ascites, gallbladder thickening, and hepatosplenomegaly. The co-occurrence of various symptoms was weighed with a heatmap diagram. We additionally correlated all clinical traits of the study participants. R software (version 4.4.2; R Foundation for Statistical Computing, Vienna, Austria) was deployed for statistical analysis and data visualization.

Results: One hundred twenty-one dengue patients were deemed eligible for this study. Their median age was 45.0 (33.0-56.0) years. Most were younger individuals (100, 83.6%) and males (100, 83.6%). Twenty-nine (24.0%) participants had severe dengue. Forty-two (34.7%) presented with some warning signs of dengue. Fifty (41.3%) participants had dengue without any warning signs. There were increased instances of gallbladder thickening (13), pleural effusion with ascites (11), and hepatosplenomegaly (19) among those with severe dengue. The median platelet counts were as follows: those with warning signs (43.5 (25.3-150.0) x 10^9^/L), those without warning signs (194.0 (178.3-210.0) x 10^9^/L), and severe dengue (25.0 (25.0-43.0) x 10^9^/L), respectively (p < 0.001). The liver enzymes are substantially increased among severe dengue patients. The co-occurrence of fever was highest with vomiting (41, 33.9%), followed by headache (39, 32.2%), body aches (36, 29.8%), and pain in the abdomen (32, 26.4%). Significant correlations were observed between durations of itching and skin rash (r = 0.96, p < 0.001), serum creatinine and urea (r = 0.82, p < 0.001), packed cell volume (PCV), and hemoglobin (r = 0.80, p < 0.001). Hospital stay was lengthened among the severe dengue patients.

Conclusion: The patients admitted with severe dengue infection experienced thrombocytopenia, raised liver enzymes, and increased hospital stays. They also had higher incidences of pleural effusion, ascites, hepatosplenomegaly, and gallbladder thickening. Fever was the most common symptom. We did not find a strong association of any clinical parameters with hospital stay.

## Introduction

Dengue fever has been spreading globally over the last two decades. The World Health Organization (WHO) forecasted a 10-fold spike in global dengue cases, from 0.5 million in 2000 to 5.2 million in 2019 [1,2]. During the COVID-19 pandemic, there was an alarming rise in dengue cases in Southeast Asian countries, i.e., India, Pakistan, and Bangladesh [3]. Rapid urbanization, high population density, and gradual climate change collectively could have contributed to these skyrocketing incidences of dengue fever in India and its neighborhood [4]. Vector-borne diseases, like dengue, are on track to become a major public health concern in India [2,4]. Dengue fever is a self-limiting condition that requires only minimum supportive care. However, in less than 1% of individuals, severe dengue symptoms such as clinical fluid buildup, shock, and multiple organ dysfunction can lead to death if not treated [3-6].

In 1997, the WHO listed three kinds of dengue infection: dengue fever, dengue hemorrhagic fever, and dengue shock syndrome [7,8]. In 2009, the classification was amended to encompass the following three types: dengue with warning signs, dengue without warning signs, and severe dengue [4,9,10]. The common symptoms of dengue infection are nausea, vomiting, skin rashes, abdominal pain, body aches, and headache. The warning signs are persistent vomiting, lethargy, restlessness, mucosal bleeding, fluid accumulation, hepatomegaly > 2 cm, and rapid decline in the platelet count. Patients with severe dengue exhibit one or more of the following symptoms: shock, respiratory distress, impaired consciousness, severe bleeding (assessed by clinician), and elevated liver enzymes [4,7,11]. Males and younger individuals are more commonly affected by dengue than females and older adults [12-15].

The incidences of hepatosplenomegaly, gallbladder thickening, pleural effusion, and ascites increase with age and severity of dengue infection [4,16-18]. The targets for the dengue virus in the liver are hepatocytes and Kupffer cells. Dengue patients often have mononuclear cell infiltrates and Councilman bodies in the portal circulation, fatty liver, hyperplasia of Kupffer cells, and hepatocyte necrosis. Such patients present with hepatomegaly and raised liver enzymes [19]. Increased vascular permeability and hepatobiliary dysfunction lead to edematous thickening of the gallbladder wall [17]. Plasma leakage in dengue patients is also manifested by pleural effusion. It is often accompanied by ascites [18].

The complete blood count (CBC) and immature cell (i.e., platelet, reticulocyte, and granulocyte) fractions show a fluctuating trend with the severity of dengue fever [20-23]. Dengue virus typically targets megakaryocytes, thereby causing thrombocytopenia. The dengue virus interferes with the translational machinery to generate the non-structural 1 (NS1) protein and fresh virions. NS1 binding to toll-like receptor 4 (TLR4) on human platelets reinforces platelet activation characterized by higher P-selectin expression, triggering platelet death [21,24]. The immature platelet fraction (IPF) symbolizes the extent of thrombopoiesis in dengue patients upon exposure to increased platelet consumption or clearance [21]. During the initial period of dengue, platelet count and IPF exhibit an inverse relationship. In severe dengue patients, slower platelet recovery makes this negative association more subtle [21,24]. C-reactive protein (CRP) is one of the acute-phase proteins synthesized from hepatocytes as an immune response to inflammatory and infectious conditions. CRP levels also indicate the severity of dengue infection [25,26].

A multitude of factors influence the prognosis and hospital stay of dengue patients. Hence, we mapped this study to evaluate and compare various clinical parameters (i.e., CBC, platelet count, IPF, liver and kidney function tests, CRP, serum lactate dehydrogenase (LDH), and ferritin levels) of the dengue patients. We also computed the incidences of gallbladder thickening, pleural effusion, ascites, and hepatosplenomegaly. Furthermore, we scrutinized the co-occurrences of all symptoms and correlated all clinical parameters of the study population.

## Materials and methods

This cross-sectional study assessed and compared different clinical markers among the dengue patients. From January to December 2024, the study was executed at the Kalinga Institute of Medical Sciences (KIMS) in Bhubaneswar, India. Before commencing the study, we got ethical approval from the Institutional Ethics Committee (KIIT/KIMS/IEC/1469/2023 dated 16.12.2023). Our study complied with institutional norms, the Declaration of Helsinki, and good clinical and laboratory practices.

Inclusion and exclusion criteria

In this study, we recruited adult patients who received admission to the medicine ward within the specified time frame with a positive NS1 report and clinical diagnosis of dengue. We excluded people below 18 years of age, patients diagnosed with chronic kidney and liver disease, and any malignancy or hematological disorders. We also excluded patients on anticoagulants, antiplatelets, granulocyte boosters, and blood transfusion therapy. We leveraged the Modified Diet for Renal Disease (MDRD) formula to compute the estimated glomerular filtration rate (eGFR) of the participants [27]. Those with chronic kidney disease and eGFR < 60 mL/min/m^²^ were not considered for the analysis.

Study procedure

We collected the patient data from their case sheets during their discharge from the hospital. The sociodemographic, i.e., age, gender, marital status, and socioeconomic status of the eligible participants, were noted. The Kuppuswamy classification was deployed to categorize the socioeconomic class of the participants [28]. We recorded the following parameters assessed just after their hospital admission: systolic blood pressure (SBP), hemoglobin, packed cell volume (PCV), platelet count, total leukocyte count (TLC), neutrophil, lymphocyte, and monocyte count, IPF, granulocyte (IGF), and reticulocyte (IRF), liver enzymes (i.e., aspartate transaminase (AST), alanine transaminase (ALT), alkaline phosphatase (ALP), and gamma-glutamyl transferase (GGT)), renal parameters (i.e., serum urea, creatinine, sodium, and potassium), serum LDH, CRP, and ferritin level. We recorded the incidences of gallbladder thickening, pleural effusion, ascites, and hepatosplenomegaly among the participants. We also noted the duration of their symptoms before their hospital visits and hospital stay.

As per the recent classification of dengue [9], we grouped all participants into the following three groups: dengue with warning signs, dengue without warning signs, and severe dengue. Recurrent vomiting, restlessness, lethargy, hepatomegaly > 2 cm, fluid accumulation, mucosal bleeding, and a sudden decline in the platelet count were regarded as warning signs of dengue infection. Patients with severe dengue presented with at least one of the following symptoms: respiratory distress, altered consciousness, shock, substantial bleeding, and raised liver enzymes [4,7,11].

Statistical analysis

This cross-sectional study was accomplished using convenience sampling. The Shapiro-Wilk test was implemented to ensure the normal distribution of the collected data. The summary statistics for the continuous data were the median and interquartile range (IQR). The categorical data was showcased through frequency and proportion. Through a Venn diagram, we portrayed the incidences of gallbladder thickening, pleural effusion, ascites, and hepatosplenomegaly among the participants. Using half-box-whisker and jitter plots, we illustrated the quantitative traits. The co-occurrence of symptoms was mapped with a heatmap diagram. A correlation plot aided us in assessing the association among all the parameters. We expressed the coefficients of correlations with a 95% confidence interval (CI). Version 4.4.2 of the R software (R Foundation for Statistical Computing, Vienna, Austria) was utilized for data computation [29]. The statistical significance was set at a p-value ≤ 0.05.

## Results

During the study period, 642 dengue patients were admitted to the medicine ward. Of them, 287 (44.7%) were below 18. Two hundred thirty-four (36.4%) subjects had incomplete details in their case sheets. The remaining 121 dengue patients with positive NS1 reports were gauged in this cross-sectional study. Fifty (41.3%) participants had dengue without warning signs. Forty-two (34.7%) showed some warning signs. The rest, 29 (24.0%), suffered from severe dengue. Table 1 illustrates the sociodemographic and clinical traits of those 121 study participants. Of them, only 21 (17.4%) participants were females. The study population had a median age of 45.0 (33.0-56.0) years. The majority of participants were married and belonged to a lower socioeconomic class. All vitals except body temperature during admission were similar among the study population regardless of the disease severity. The duration of hospitalization was prolonged in the patients with severe dengue. The incidences of gallbladder thickening, pleural effusion, ascites, and hepatosplenomegaly were pronounced among the patients with severe dengue.

**Table 1:** Demographic and clinical parameters of the study participants The median (IQR) was the summary statistics for the continuous data. Frequency and proportion were employed to express the categorical data. All details noted (except for the duration of hospitalization) were the initial data after hospital admission. IQR: interquartile range, SpO₂: oxygen saturation, BP: blood pressure.

Parameters	Total (n = 121)	Dengue with warning signs (n = 42)	Dengue without warning signs (n = 50)	Severe dengue (n = 29)	p-value
Age (years)	45.0 (33.0-56.0)	41.0 (33.0-57.5)	46.5 (33.0-57.3)	45.0 (35.0-52.0)	0.84
Elderly (Age > 60 years)	21 (17.4%)	10 (23.8%)	8 (16.0%)	3 (10.3%)	0.17
Gender					
Male	100 (82.6%)	36 (85.7%)	45 (90.0%)	19 (65.5%)	0.27
Female	21 (17.4%)	6 (14.3%)	5 (10.0%)	10 (34.5%)	
Marital status					
Married	109 (90.1%)	38 (90.5%)	46 (92.0%)	25 (86.2%)	< 0.001
Unmarried	9 (7.4%)	3 (7.1%)	2 (4.0%)	4 (13.8%)	
Divorced/widowed	3 (2.5%)	1 (2.4%)	2 (4.0%)	0	
Socioeconomic status					
Low	78 (64.5%)	28 (66.7%)	37 (74.0%)	13 (44.8%)	< 0.001
Lower middle	33 (27.2%)	12 (28.6%)	11 (22.0%)	10 (34.5%)	
Upper middle	10 (8.3%)	2 (4.7%)	2 (4.0%)	6 (20.7%)	
Respiratory rate (per minute)	18 (16-20)	18 (16-20)	18 (16-20)	18 (16-20)	1
Pulse rate (per minute)	84 (78-96)	84 (80-96)	82 (78-94)	88 (76-92)	0.96
Systolic BP (mm of Hg)	118 (110-128)	116 (110-128)	119 (110-128)	120 (110-130)	0.91
Diastolic BP (mm of Hg)	72 (70-80)	71 (70-80)	74 (70-80)	72 (70-80)	0.99
SpO₂	97.0 (96.0-98.0)	97.0 (95.5-98.5)	97.0 (96.0-97.5)	96.5 (96.0-97.0)	0.93
Body temperature (^°^F)	99.9 (98.6-101.0)	99.0 (98.4-100.1)	99.7 (98.7-100.7)	101.3 (100.6-102.0)	0.03
Icterus	9 (7.4%)	2 (4.7%)	4 (8.0%)	3 (10.3%)	0.37
Positive tourniquet test	3 (2.5%)	1 (2.4%)	0	2 (6.9%)	0.89
Duration of hospitalization (in days)	6 (4-8)	6 (5-7)	4 (4-5)	10 (9-12)	< 0.001
Gallbladder thickening	28 (23.1%)	9 (21.4%)	6 (12.0%)	13 (44.8%)	< 0.001
Pleural effusion and ascites	20 (16.5%)	6 (14.3%)	3 (6.0%)	11 (37.9%)	0.04
Hepatosplenomegaly	38 (31.4%)	12 (28.6%)	7 (14.0%)	19 (65.5%)	0.01

**Figure 1:**
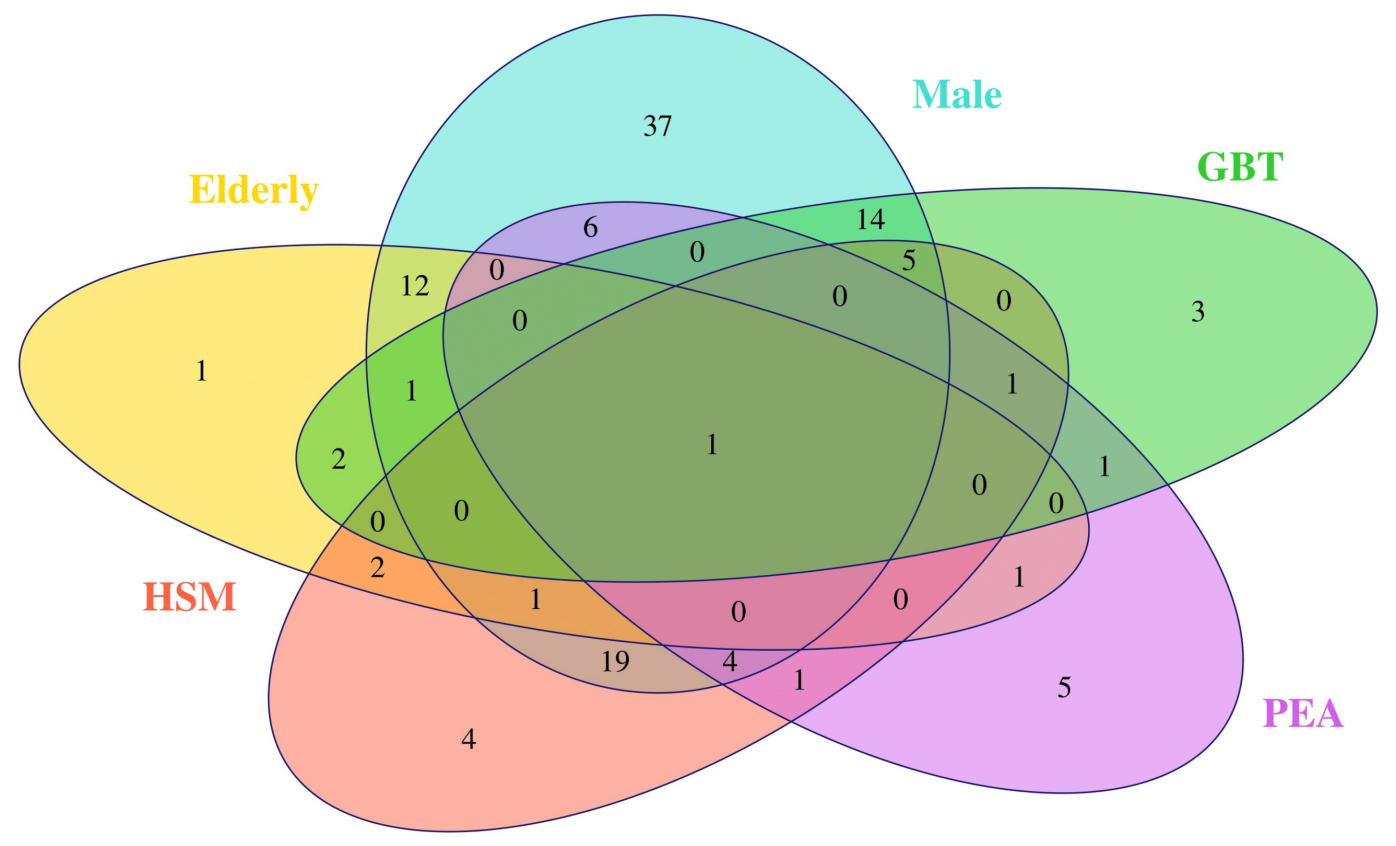
Venn diagram showing male and elderly participants with various findings The Venn diagram depicts the number of male and elderly participants with dengue exhibiting various features. HSM: hepatosplenomegaly, PEA: pleural effusion with ascites, GBT: gallbladder thickening.

The Venn diagram in Figure 1 illustrates the incidences of hepatosplenomegaly, pleural effusion with ascites, and gallbladder thickening among the elderly male subjects. Our study entailed 21 (17.4%) elderly and 100 (82.6%) male participants. The most common finding in our study was hepatosplenomegaly (38, 31.4%), followed by gallbladder thickening (28, 23.1%) and pleural effusion with ascites (20, 16.5%). Only 15 (12.4%) elderly males were present in this study. Of them, only one (0.8%) had all three above-mentioned features.

**Figure 2:**
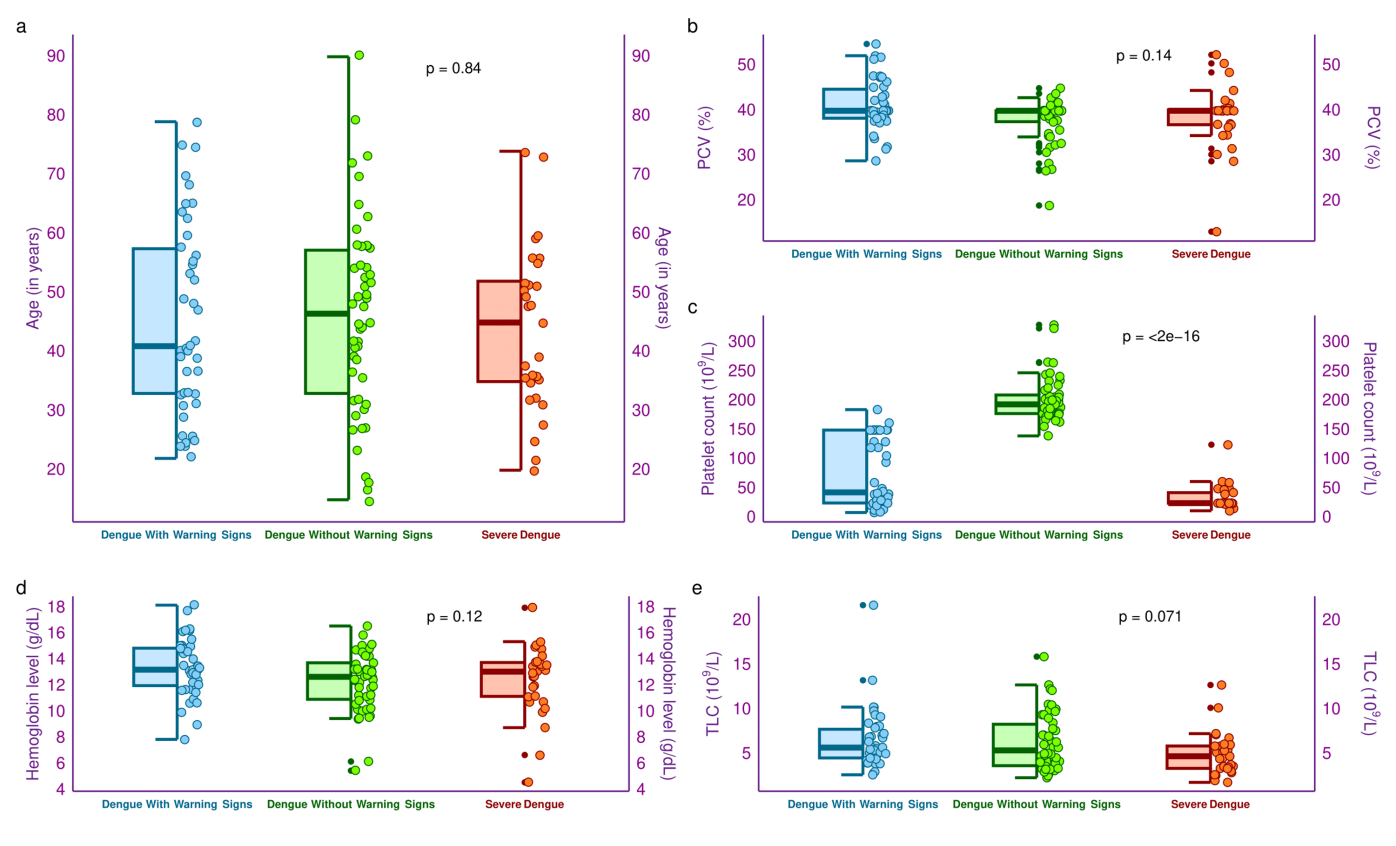
Age and hematological parameters of the study participants The half-box-whisker and jitter plots illustrate the study participants' age distribution and hematological parameters. The x-axis denotes the severity of dengue. PCV: packed cell volume, TLC: total leukocyte count.

The half-box-whisker and jitter plots in Figure 2 portray the study participants' age distribution and the hematological parameters (i.e., PCV, platelet count, hemoglobin level, and TLC). The median ages for the participants who suffered from dengue with warning signs, dengue without warning signs, and severe dengue were 41.0 (33.0-57.5) years, 46.5 (33.0-57.3) years, and 45.0 (35.0-52.0) years, respectively (p = 0.84). The median PCV values for the three groups were 40.0 (38.4-44.8)%, 40.0 (37.6-40.0)%, and 40.0 (36.9-40.2)%, respectively (p = 0.14). The median platelet counts were 43.5 (25.3-150.0) x 10^9^/L, 194.0 (178.3-210.0) x 10^9^/L, and 25.0 (25.0-43.0) x 10^9^/L, respectively (p < 0.001). The median blood hemoglobin values of the three study groups were 13.3 (12.0-14.9) gm/dL, 12.7 (11.0-13.8) gm/dL, and 13.1 (11.2-13.8) gm/dL, respectively (p = 0.12). The median TLC values of the three study groups were 5.8 (4.6-7.8) x 10^9^/L, 5.4 (3.7-8.4) x 10^9^/L, and 4.8 (3.4-5.9) x 10^9^/L, respectively (p = 0.071). All the intergroup analyses revealed non-significant differences except the platelet count.

**Figure 3:**
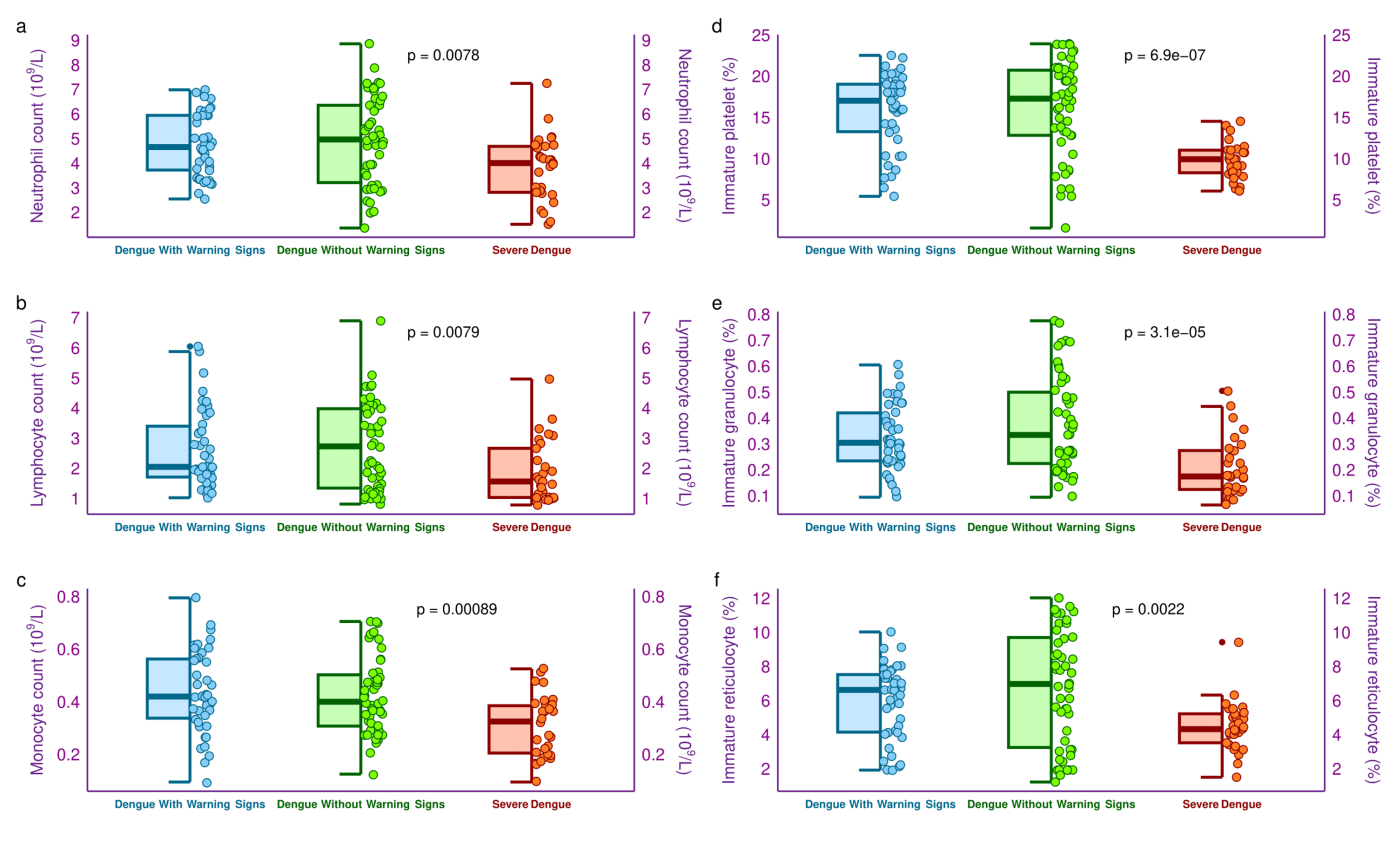
Hematological counts and fractions of the study population The half-box-whisker and jitter plots portray haematological counts and fractions of the study participants. The x-axis denotes the severity of dengue.

The half-box-whisker and jitter plots in Figure 3 portray the neutrophil, lymphocyte, and monocyte counts, as well as IPF, IGF, and IRF of the three study groups' participants. The median neutrophil counts of the three study groups were 4.7 (3.8-6.0) x 10^9^/L, 5.0 (3.3-6.4) x 10^9^/L, and 4.1 (2.9-4.7) x 10^9^/L, respectively (p = 0.008). Similarly, the median lymphocyte counts of the three study groups were 2.1 (1.8-3.4) x 10^9^/L, 2.8 (1.4-4.0) x 10^9^/L, and 1.6 (1.1-2.7) x 10^9^/L, respectively (p = 0.008). The median monocyte counts of the three study groups were 0.43 (0.34-0.57) x 10^9^/L, 0.41 (0.31-0.51) x 10^9^/L, and 0.33 (0.21-0.39) x 10^9^/L, respectively (p < 0.001). The median IPF values of the three study groups were 17.2 (13.4-19.2)%, 17.5 (13.0-20.9)%, and 10.1 (8.5-11.2)%, respectively (p < 0.001). Similarly, the median IGF values of the three groups were 0.31 (0.24-0.43)%, 0.34 (0.23-0.51)%, and 0.18 (0.13-0.28)%, respectively (p < 0.001). The median IRF values of the three study groups were 6.7 (4.3-7.6)%, 7.1 (3.3-9.8)%, and 4.4 (3.6-5.3)%, respectively (p = 0.002). The intergroup comparisons yielded statistically significant differences for all parameters.

**Figure 4:**
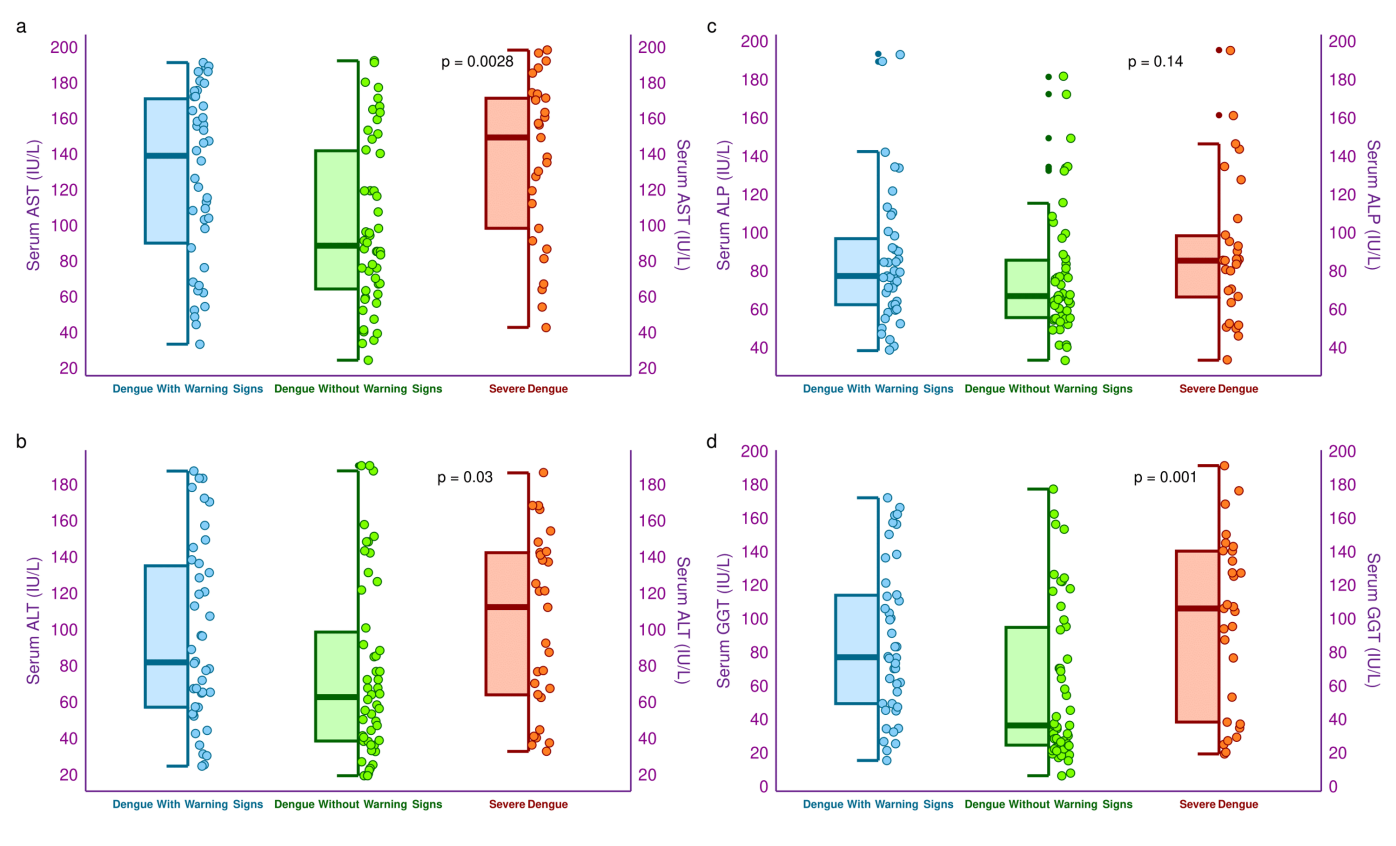
Hepatic enzymes of the study participants The half-box-whisker and jitter plots showcase the hepatic enzymes of the study participants. The x-axis presents the severity of dengue. AST: aspartate transaminase, ALT: alanine transaminase, ALP: alkaline phosphatase, GGT: gamma-glutamyl transferase.

The half-box-whisker and jitter plots in Figure 4 showcase the liver enzyme (i.e., AST, ALT, ALP, and GGT) levels of the three study groups’ participants. The median AST levels of the three study groups were 139.7 (90.7-171.7) IU/L, 89.3 (65.0-142.5) IU/L, and 150.0 (99.0-172.0) IU/L, respectively (p = 0.003). Similarly, the median ALT levels of the three study groups were 82.5 (57.8-135.8) IU/L, 63.4 (39.1-99.2) IU/L, and 113.0 (64.6-143.0) IU/L, respectively (p = 0.03). The median ALP levels of the three study groups were 78.0 (63.0-97.5) IU/L, 67.5 (56.3-86.3) IU/L, and 86.0 (67.0-99.0) IU/L, respectively (p = 0.14). The median GGT levels of the three study groups were 77.7 (50.0-114.7) IU/L, 37.0 (25.3-95.5) IU/L, and 106.8 (39.0-141.0) IU/L, respectively (p = 0.001). The between-group analyses revealed statistically significant differences for all hepatic parameters except ALP.

**Figure 5:**
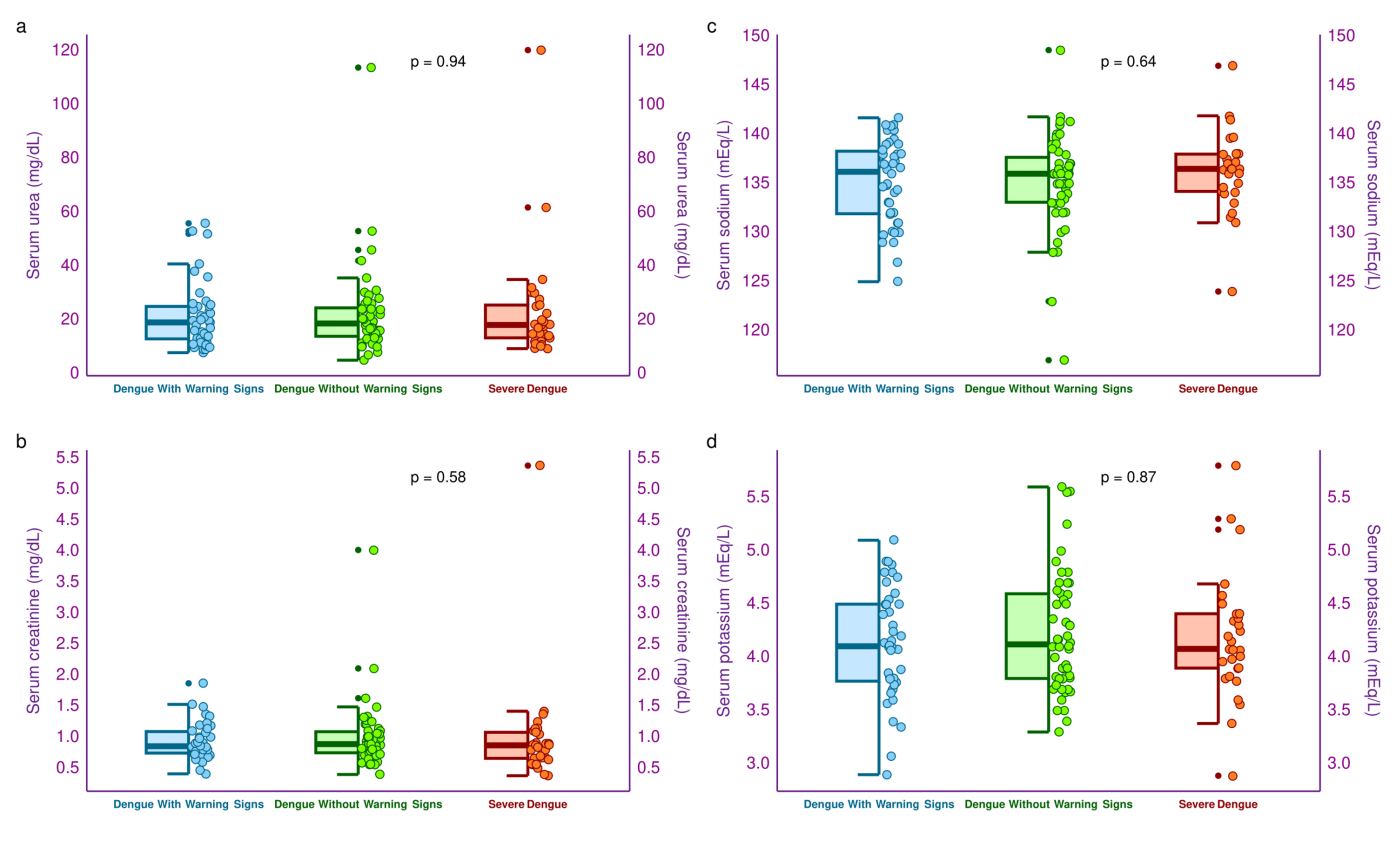
Renal parameters of the study participants The half-box-whisker and jitter plots showcase the renal parameters of the study participants. The x-axis denotes the severity of dengue.

The half-box-whisker and jitter plots in Figure 5 illustrate the renal parameters (i.e., blood urea, serum creatinine, serum sodium, and serum potassium) of the three study groups’ participants. The median blood urea levels of the three study groups were 19.1 (12.9-25.0) mg/dL, 18.7 (13.9-24.5) mg/dL, and 18.1 (13.3-25.5) mg/dL, respectively (p = 0.94). The median serum creatinine levels of the three study groups were 0.86 (0.75-1.09) mg/dL, 0.89 (0.75-1.09) mg/dL, and 0.87 (0.66-1.08) mg/dL, respectively (p = 0.58). The median serum sodium levels of the three study groups were 136.2 (131.9-138.3) mEq/L, 136.0 (133.1-137.7) mEq/L, and 136.5 (134.2-138.0) mEq/L, respectively (p = 0.64). Similarly, median serum potassium levels of the three groups were 4.1 (3.8-4.5) mEq/L, 4.1 (3.8-4.6) mEq/L, and 4.1 (3.9-4.4) mEq/L, respectively (p = 0.64). The intergroup comparisons did not provide any significant difference for the renal parameters.

**Figure 6:**
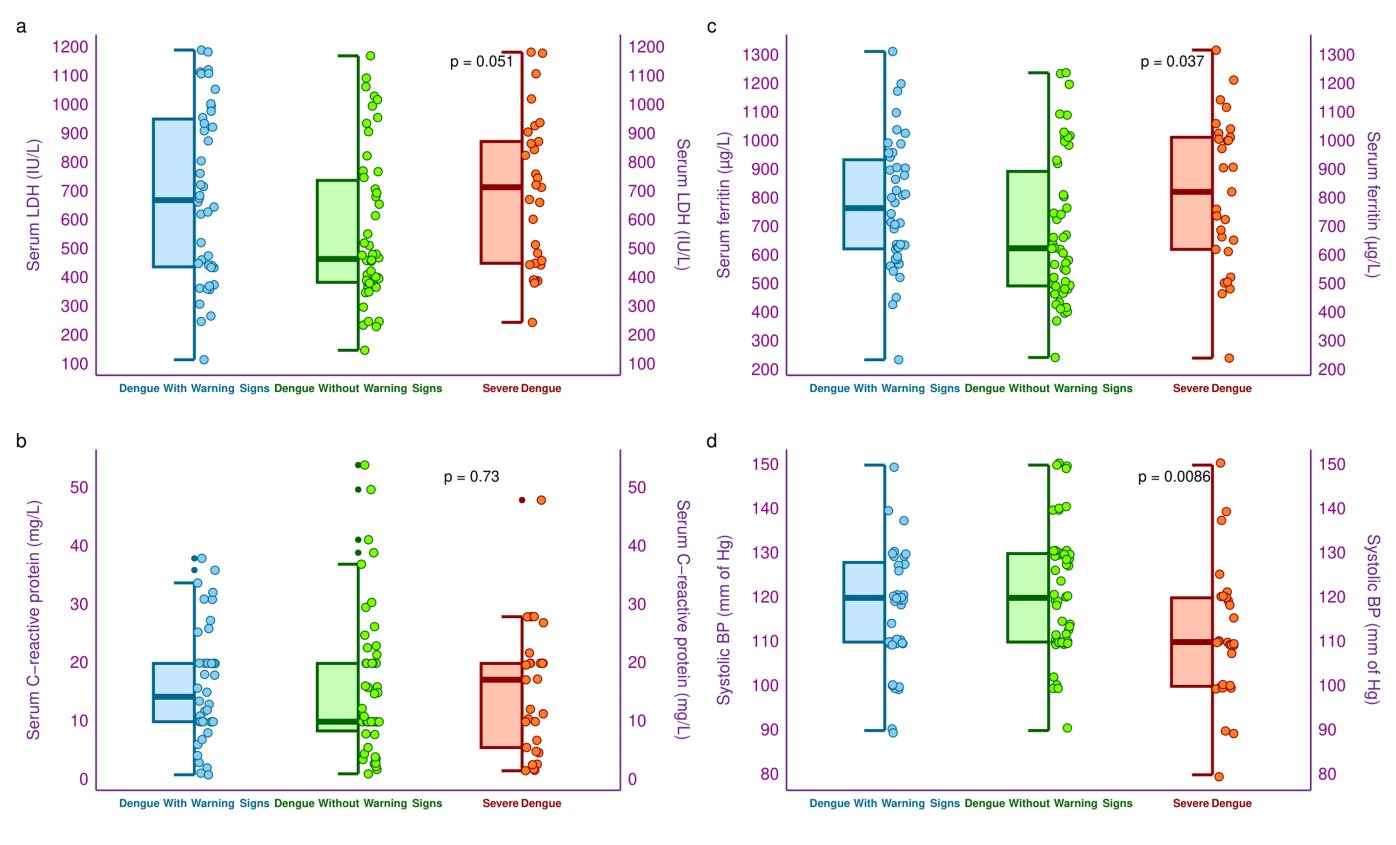
Serum LDH, CRP, ferritin, and systolic BP of the study population The half-box-whisker and jitter plots show the study participants' serum LDH, CRP, ferritin, and systolic BP. The x-axis denotes the severity of dengue. CRP: C-reactive protein, LDH: lactate dehydrogenase, BP: blood pressure.

The half-box-whisker and jitter plots in Figure 6 illustrate the serum LDH, CRP, ferritin, and systolic BP levels of the three study groups' participants. The median serum LDH levels of the three study groups were 671.3 (439.6-952.9) IU/L, 466.9 (385.8-740.0) IU/L, and 716.0 (452.0-875.0) IU/L, respectively (p = 0.051). The median serum CRP levels of the three study groups were 14.3 (10.0-20.0) mg/L, 10.0 (8.4-20.0) mg/L, and 17.2 (5.6-20.0) mg/L, respectively (p = 0.73). The median serum ferritin levels of the three study groups were 767.3 (624.8-936.4) µg/L, 627.0 (495.3-895.8) µg/L, and 824.0 (623.0-1015.0) µg/L, respectively (p = 0.037). Similarly, the median values of systolic BP of the three groups were 119.0 (110.5-128.0) mm of Hg, 122.0 (112.0-128.0) mm of Hg, and 114.0 (102.0-122.0) mm of Hg, respectively (p = 0.009).

**Figure 7:**
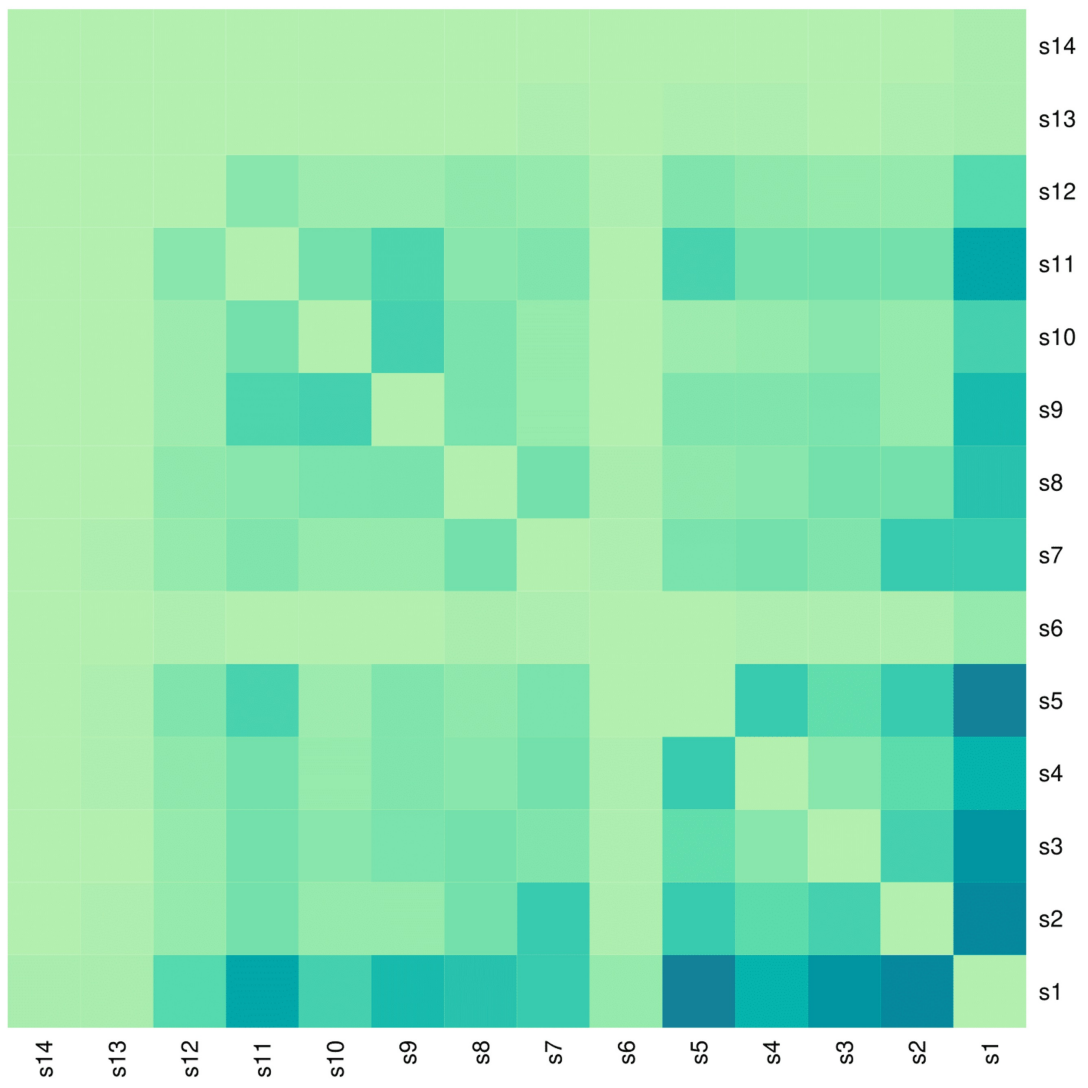
Co-occurrence of dengue symptoms in the study population The heatmap diagram portrays the co-occurrence of dengue symptoms among the participants. Both axes represent the 14 symptoms noted in the study population. The bottom-left and top-right portions of the diagram are diagonally symmetrical. The lighter shade of blue indicates a lower degree of co-occurrence of symptoms. Darker shades of green denote a higher degree of co-occurrence of symptoms. s1: fever, s2: headache, s3: body aches, s4: nausea, s5: vomiting, s6: dizziness, s7: retro-orbital pain, s8: arthralgia, s9: skin rash, s10: itching, s11: abdominal pain, s12: loose motion, s13: oliguria, s14: bleeding manifestation.

The heatmap diagram in Figure 7 portrays the co-occurrence of symptoms experienced by our 121 study participants. The top-right and bottom-left portions of the heatmap diagram are diagonally symmetrical. The highest co-occurrence was observed between fever and vomiting (41, 33.9%), followed by fever and headache (39, 32.2%), fever and body ache (36, 29.8%), fever and pain abdomen (32, 26.4%), fever and nausea (27, 22.3%), fever and skin rash (24, 19.8%), fever and retro-orbital pain (19, 15.7%), headache and retro-orbital pain (19, 15.7%), headache and vomiting (19, 15.7%), nausea and vomiting (19, 15.7%), headache and body ache (17, 14.0%), skin rash and itching (17, 14.0%), vomiting and pain abdomen (16, 13.2%), and skin rash and pain abdomen (15, 12.4%).

**Figure 8:**
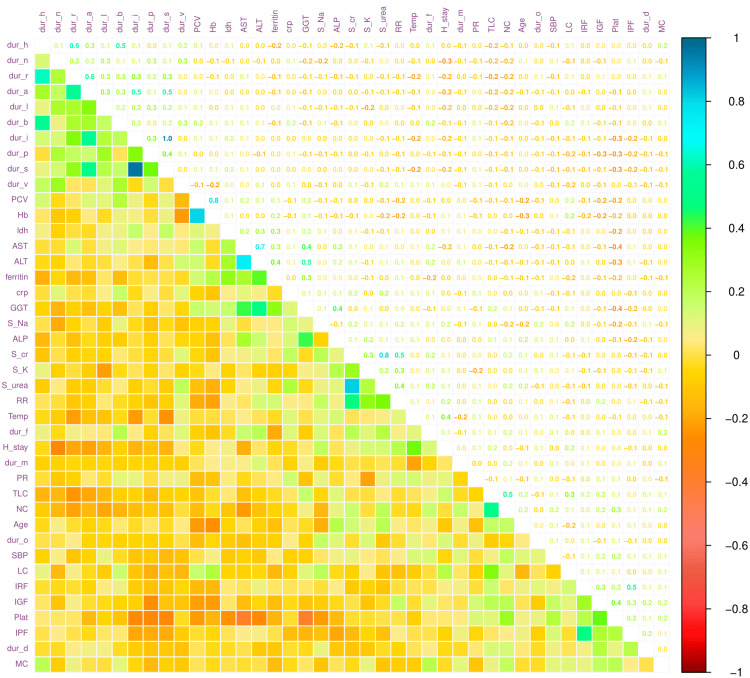
Correlation of various parameters of the study participants The correlation plot portrays the degree of association among various parameters of the study participants. The correlation coefficients span from +1 (indicating a strongly positive correlation) to -1 (indicating a strongly negative correlation). Hb: blood hemoglobin, Plat: platelet count, PCV: packed cell volume, TLC: total leukocyte count, NC: neutrophil count, LC: lymphocyte count, MC: monocyte count, IPF: immature platelet fraction, IGF: immature granulocyte fraction, IRF: immature reticulocyte fraction, AST: aspartate transaminase, ALT: alanine transaminase, ALP: alkaline phosphatase, GGT: gamma-glutamyl transferase, S_urea: serum urea, S_cr: serum creatinine, S_Na: serum sodium, S_K: sodium potassium, ldh: lactate dehydrogenase, crp: C-reactive protein, dur_f: duration of fever, dur_h: duration of headache, dur_b: duration of body aches, dur_n: duration of nausea, dur_v: duration of vomiting, dur_d: duration of dizziness, dur_r: duration of retro-orbital pain, dur_a: duration of arthralgia, dur_s: duration of skin rash, dur_i: duration of itching, dur_p: duration of abdominal pain, dur_l: duration of loose motion, dur_o: duration of oliguria, dur_m: duration of bleeding manifestation, PR: pulse rate, RR: respiratory rate, H_stay: hospital stay, SBP: systolic blood pressure, Temp: body temperature.

Figure 8 portrays the correlation coefficients among the various parameters assessed in this study. There were positive correlations between durations of skin rash and itching (r = 0.96, 95% CI = 0.95-0.97, p < 0.001), serum urea and serum creatinine (r = 0.82, 95% CI = 0.75-0.87, p < 0.001), hemoglobin and PCV (r = 0.80, 95% CI = 0.73-0.86, p < 0.001), AST and ALT (r = 0.73, 95% CI = 0.64-0.81, p < 0.001), durations of retro-orbital pain and headache (r = 0.61, 95% CI = 0.49-0.71, p < 0.001), durations of retro-orbital pain and arthralgia (r = 0.56, 95% CI = 0.43-0.67, p < 0.001), TLC and neutrophil count (r = 0.51, 95% CI = 0.37-0.63, p < 0.001), and ALT and GGT (r = 0.48, 95% CI = 0.33-0.61, p < 0.001). The duration of hospitalization was positively associated with the body temperature measured during the admission (r = 0.37, 95% CI = 0.21-0.52, p < 0.001). There were no strong negative correlations in our study. Moreover, we did not find any variables strongly associated with hospital stay.

## Discussion

This cross-sectional study analyzed 121 (18.9%) of 642 dengue patients admitted to the medicine ward. The study participants had a median age of 45.0 (33.0-56.0) years. Among the 121 participants, 50 had dengue without warning signs, 42 exhibited one or more warning signs, and 29 were diagnosed with severe dengue. Most participants were males (100, 83.6%) and younger individuals (100, 83.6%). The patients with severe dengue experienced longer hospital stays. They also exhibited high rates of hepatosplenomegaly, pleural effusion, ascites, and gallbladder thickening. Our findings concorded with that of the studies by Htun et al. [4] and Ibrahim et al. [17].

Thrombocytopenia was observed among the participants, with warning signs and severe dengue. It was more pronounced among those with severe dengue. It could be attributed to NS1 overactivity in severe dengue patients [21,24]. The CBC revealed that severe dengue patients had lower counts of neutrophils, lymphocytes, and monocytes than the other two groups. Our observations matched two recent studies [22,23]. The rate of thrombopoiesis after exposure to platelet apoptosis is quantified with IPF [21]. Platelet count and IPF demonstrate an inverse association during the initial stages of dengue. Higher apoptosis rates and slower rates of thrombopoiesis among severe dengue patients lead to reduced IPF [21]. Reduced values of IGF and IRF among severe dengue patients could be explained by their faster clearance and slower genesis rates [24,30,31].

The liver enzymes were significantly elevated among the patients with severe dengue, as contrasted with the other two groups. As the liver is a major target for the dengue virus, hepatomegaly and raised liver enzymes were more evident among those with severe dengue. A recent study by Chia et al. [32] supported our findings. Kidney functions were similar across the three groups, indicating a lower impact of the dengue virus on the kidneys than the liver. Our study participants had similar levels of serum LDH. Kutsuna et al. [26] advocated that serum LDH levels increase after red blood cells (RBC) are broken down. Normal hemoglobin levels of most of our study population could explain the serum LDH levels. CRP levels are predominantly elevated among patients with severe dengue. It could be traced to their hepatic origin and liver involvement in dengue fever [25,32]. We observed higher levels of serum ferritin among the severe dengue patients. The study by Suresh et al. [33] corroborated our findings.

We gauged the clinical parameters of dengue patients admitted to our hospital in the entire calendar year of 2024. The major plus of our study was data visualization through Venn diagrams, heatmaps, and correlation plots. We could not find any similar studies on dengue assessed through these plots. Therefore, we could not deduce any similarities or differences of these plots with any of the previous studies on dengue. Our study had some limitations as well. First, we could not analyze more patients owing to a lack of information in their case sheets. Second, we did not assess the impact of comorbidities and concomitant medications on the dengue patients' clinical traits and hospital stay. Third, we neither analyzed the follow-up investigations nor followed them up after discharge.

## Conclusions

The patients with severe dengue infection had pronounced thrombocytopenia, heightened liver enzymes, and prolonged hospitalization. They also experienced increased incidences of hepatosplenomegaly, pleural effusion, ascites, and gallbladder thickening. They also exhibited lower counts of neutrophils, monocytes, and lymphocytes. The immature cell fractions, i.e., IPF, IGF, and IRF, were on the lower side for those with severe dengue. Among the various symptoms of dengue infection, fever was the most common. We did not observe any clinical parameter to be strongly associated with the duration of hospitalization. We urge prospective studies with a heterogeneous population to evaluate various clinical traits of dengue infection and their associations.

## References

[1] Carlson CJ, Boyce MR, Dunne M (2023). The World Health Organization's disease outbreak news: a retrospective database. PLOS Glob Public Health.

[2] Singh PS, Chaturvedi HK (2024). Socio-ecological predictors of dengue in high incidence area of Delhi, India. Sci Rep.

[3] Khan S, Akbar SM, Yahiro T, Mahtab MA, Kimitsuki K, Hashimoto T, Nishizono A (2022). Dengue Infections during COVID-19 period: reflection of reality or elusive data due to effect of pandemic. Int J Environ Res Public Health.

[4] Htun TP, Xiong Z, Pang J (2021). Clinical signs and symptoms associated with WHO severe dengue classification: a systematic review and meta-analysis. Emerg Microbes Infect.

[5] Sharma H, Ilyas A, Chowdhury A (2022). Does COVID-19 lockdowns have impacted on global dengue burden? A special focus to India. BMC Public Health.

[6] Tsheten T, Gray DJ, Clements AC, Wangdi K (2021). Epidemiology and challenges of dengue surveillance in the WHO South-East Asia Region. Trans R Soc Trop Med Hyg.

[7] John KJ, Gunasekaran K, Prasad JD (2019). Predictors of major bleeding and mortality in dengue infection: a retrospective observational study in a tertiary care centre in south India. Interdiscip Perspect Infect Dis.

[8] Rigau-Pérez JG, Clark GG, Gubler DJ, Reiter P, Sanders EJ, Vorndam AV (1998). Dengue and dengue haemorrhagic fever. Lancet.

[9] Morra ME, Altibi AM, Iqtadar S (2018). Definitions for warning signs and signs of severe dengue according to the WHO 2009 classification: systematic review of literature. Rev Med Virol.

[10] World Health Organization (2015). National guidelines for clinical management of dengue fever. https://ncvbdc.mohfw.gov.in/Doc/National%20Guidelines%20for%20Clinical%20Management%20of%20Dengue%20Fever%202023.pdf.

[11] Thein TL, Gan VC, Lye DC, Yung CF, Leo YS (2013). Utilities and limitations of the World Health Organization 2009 warning signs for adult dengue severity. PLoS Negl Trop Dis.

[12] Chakravarti A, Roy P, Malik S, Siddiqui O, Thakur P (2016). A study on gender-related differences in laboratory characteristics of dengue fever. Indian J Med Microbiol.

[13] Kumar M, Verma RK, Mishra B (2020). Prevalence of dengue fever in western Uttar Pradesh, India: a gender-based study. Int J Appl Basic Med Res.

[14] Chang K, Huang CH, Chen TC, Lin CY, Lu PL, Chen YH (2021). Clinical characteristics and risk factors for intracranial hemorrhage or infarction in patients with dengue. J Microbiol Immunol Infect.

[15] Thai KT, Nishiura H, Hoang PL (2011). Age-specificity of clinical dengue during primary and secondary infections. PLoS Negl Trop Dis.

[16] de Oliveira Neto EG, Nascimento DD, Bertoncini TV, Lopes AA, Bezerra AS, Soares MV (2024). The role of the radiologist in the dengue endemic: a pictorial essay. Radiol Bras.

[17] Ibrahim MA, Hamzah SS, Md Noor J, Mohamad MI, Mokhtar MF, Isa MR, Abdul Rani MF (2022). The association of ultrasound assessment of gallbladder wall thickness with dengue fever severity. Ultrasound J.

[18] Kaagaard MD, Matos LO, Evangelista MV (2023). Frequency of pleural effusion in dengue patients by severity, age and imaging modality: a systematic review and meta-analysis. BMC Infect Dis.

[19] Samanta J, Sharma V (2015). Dengue and its effects on liver. World J Clin Cases.

[20] Abeysuriya V, Seneviratne SL, de Mel P (2022). The immature platelet fraction, a predictive tool for early recovery from dengue-related thrombocytopenia: a prospective study. Trans R Soc Trop Med Hyg.

[21] Khazali AS, Hadrawi WH, Ibrahim F, Othman S, Nor Rashid N (2024). Thrombocytopenia in dengue infection: mechanisms and a potential application. Expert Rev Mol Med.

[22] Haider RZ, Khan NA, Urrechaga E, Shamsi TS (2021). Mature and immature/activated cells fractionation: time for a paradigm shift in differential leucocyte count reporting?. Diagnostics (Basel).

[23] Ananda Rao A, U RR, Gosavi S, Menon S (2020). Dengue fever: prognostic insights from a complete blood count. Cureus.

[24] de Azeredo EL, Monteiro RQ, de-Oliveira Pinto LM (2015). Thrombocytopenia in dengue: interrelationship between virus and the imbalance between coagulation and fibrinolysis and inflammatory mediators. Mediators Inflamm.

[25] Vuong NL, Le Duyen HT, Lam PK (2020). C-reactive protein as a potential biomarker for disease progression in dengue: a multi-country observational study. BMC Med.

[26] Kutsuna S, Hayakawa K, Kato Y (2014). The usefulness of serum C-reactive protein and total bilirubin levels for distinguishing between dengue fever and malaria in returned travelers. Am J Trop Med Hyg.

[27] Michels WM, Grootendorst DC, Verduijn M, Elliott EG, Dekker FW, Krediet RT (2010). Performance of the Cockcroft-Gault, MDRD, and new CKD-EPI formulas in relation to GFR, age, and body size. Clin J Am Soc Nephrol.

[28] Khairnar MR, Wadgave U, Shimpi PV (2017). Kuppuswamy's socio-economic status scale: a revision of occupation and income criteria for 2016. Indian J Pediatr.

[29] (2025). R: A language and environment for statistical computing, Vienna, Austria. https://www.r-project.org/.

[30] Chaloemwong J, Tantiworawit A, Rattanathammethee T, Hantrakool S, Chai-Adisaksopha C, Rattarittamrong E, Norasetthada L (2018). Useful clinical features and hematological parameters for the diagnosis of dengue infection in patients with acute febrile illness: a retrospective study. BMC Hematol.

[31] Kularatnam GA, Jasinge E, Gunasena S, Samaranayake D, Senanayake MP, Wickramasinghe VP (2019). Evaluation of biochemical and haematological changes in dengue fever and dengue hemorrhagic fever in Sri Lankan children: a prospective follow up study. BMC Pediatr.

[32] Chia PY, Thein TL, Ong SW, Lye DC, Leo YS (2020). Severe dengue and liver involvement: an overview and review of the literature. Expert Rev Anti Infect Ther.

[33] Suresh SC, Hanumanthaiah R, Ramakrishna C (2020). Serum ferritin as a prognostic indicator in adult dengue patients. Am J Trop Med Hyg.

